# Capturing phenotypes for precision medicine

**DOI:** 10.1101/mcs.a000372

**Published:** 2015-10

**Authors:** Peter N. Robinson, Christopher J. Mungall, Melissa Haendel

**Affiliations:** 1 Institute for Medical Genetics and Human Genetics, Charité-Universitätsmedizin Berlin, 10117 Berlin, Germany;; 2 Max Planck Institute for Molecular Genetics, 14195 Berlin, Germany;; 3 Berlin Brandenburg Center for Regenerative Therapies (BCRT), Charité-Universitätsmedizin Berlin, 13353 Berlin, Germany;; 4 Institute for Bioinformatics, Department of Mathematics and Computer Science, Freie Universität Berlin, 14195 Berlin, Germany;; 5 Lawrence Berkeley National Laboratory, Berkeley, California 94720, USA;; 6 Oregon Health and Science University, Portland, Oregon 97239, USA

## Abstract

Deep phenotyping followed by integrated computational analysis of genotype and phenotype is becoming ever more important for many areas of genomic diagnostics and translational research. The overwhelming majority of clinical descriptions in the medical literature are available only as natural language text, meaning that searching, analysis, and integration of medically relevant information in databases such as PubMed is challenging. The new journal *Cold Spring Harbor Molecular Case Studies* will require authors to select Human Phenotype Ontology terms for research papers that will be displayed alongside the manuscript, thereby providing a foundation for ontology-based indexing and searching of articles that contain descriptions of phenotypic abnormalities—an important step toward improving the ability of researchers and clinicians to get biomedical information that is critical for clinical care or translational research.

A phenotypic abnormality is defined in medical settings as a deviation from normal morphology, physiology, or behavior, and good phenotyping is a cornerstone of a doctor's daily work (Baynam et al. 2015). Next-generation sequencing, proteomics, and metabolomics data as well as information technologies are bringing about a paradigm shift in translational research and also clinical care. Although the coming era will allow physicians and patients to access large-scale data with the potential to stratify and thereby improve medical treatment, the ability to find correct and up-to-date information with sufficiently detailed and accurate phenotypic descriptions will be essential to exploit this data to its fullest (Fernald et al. 2011). In this article, we will discuss the role of deep phenotyping in translational research and the challenges in using this information for integrated computational analysis of “omics” data in the medical arena, as well as the application of the Human Phenotype Ontology (HPO) as a standardized, comprehensive nomenclature for disease-associated phenotypic abnormalities.

## DEEP PHENOTYPING

Deep phenotyping can be defined as the precise and comprehensive analysis of phenotypic abnormalities in which the individual components of the phenotype are observed and described, generally in such a way as to be computationally accessible. Precision medicine has the goal of providing the best available care for each patient based on stratification into disease subclasses for which there is a common biological basis. The comprehensive discovery of such subclasses, as well as the translation of this knowledge into clinical care, will depend critically upon computational resources to capture, store, and exchange deep phenotypic data. Further, sophisticated algorithms to integrate deep phenotype data with genomic variation and additional clinical information will be required in support of precision medicine (Robinson 2012).

## TEXT MINING PHENOTYPE DATA

A “traditional” method of retrieving phenotype data from the medical literature or Electronic Health Records for computational analysis is text mining. However, the overwhelming majority of clinical descriptions in the medical literature are simply natural language text, and thus automated searching, analysis, and integration of medical information from databases such as PubMed remains challenging (Taboada et al. 2014). For instance, in the phrase “short long bones” the word “long” is part of the concept long bone (e.g., the femur and the humerus are long bones, but the skull is not). However, in the phrase “long metacarpal,” the word “long” is used to denote an abnormally increased length of metacarpal bones. Similarly, the medical literature abounds in phrases such as “the patient was still ambulatory after 25 years,” or “segmentation defects appear to affect L4-S1” that can be very evocative to trained physicians but next to impossible to interpret correctly by text mining. Therefore, although sophisticated concept recognition algorithms have been developed to improve the results of text mining for phenotype data (Groza et al. 2015), it remains difficult to extract the clinical information from an article in a correct and comprehensive fashion. The need for improved online search tools to find and analyze publications on patients with similar clinical characteristics is especially critical and challenging for rare diseases, where publications of large series are scarce.

There are several current clinical nomenclatures for phenotyping such as Medical Subject Headings (MeSH), the ICD-10, the National Cancer Institute's (NCI) Thesaurus, SNOMED CT, and the United Medical Language System (UMLS). However, phenotypic concepts are covered inconsistently and incompletely in most currently used clinical terminologies (Winnenburg and Bodenreider 2014). For example, MeSH provides little semantic distinction between disease entities and phenotypic manifestations of diseases. For instance, even though the MeSH category C is described as comprising Diseases, it contains many entries that describe phenotypic features of diseases rather than an actual disease entities, such as Cheyne–Stokes Respiration (MeSH: D002639), which is an abnormal pattern of breathing that can be observed in diseases such as central sleep apnea syndrome. Even with these clinical nomenclatures, it can be difficult for clinicians and researchers to find relevant biomedical articles on a phenotypic abnormality using PubMed or Google Scholar.

## A NEW APPROACH TO CAPTURING PHENOTYPES

**Figure 1.**
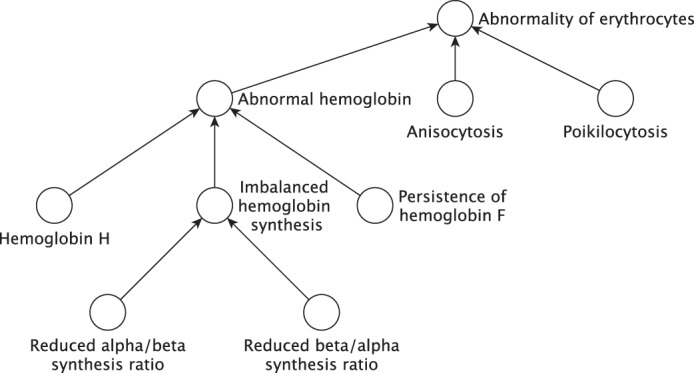
An excerpt of the hierarchical structure of the Human Phenotype Ontology. The terms of the HPO are arranged in a subclass hierarchy. For instance, any patient annotated to the HPO term “Reduced beta/alpha synthesis ratio” (HP:0011906) can also be said to have “Imbalanced hemoglobin synthesis” (HP:0005560), “Abnormal hemoglobin” (HP:0011902), and so on. Note that when selecting HPO terms for *Cold Spring Harbor Molecular Case Studies* submissions, authors may select leaf terms (i.e., the most specific terms possible). For example, “Hemoglobin H” is a leaf team, but “Abnormal hemoglobin” is not.

To overcome the limitations above, a structured, comprehensive, and well-defined phenotyping terminology is needed. The Human Phenotype Ontology (HPO), available at www.human-phenotype-ontology.org, provides a set of more than 11,000 terms describing human phenotypic abnormalities. The HPO provides both a set of terms that describe concepts of human phenotypes as well as a logical (computational) representation of the interrelationships between the terms. The HPO is arranged as a hierarchy with the most specific terms being at the greatest distance from the root term (Fig. 1). A recent study comparing HPO with alternate terminologies found that the ICD-10 covered 9%, the NCI thesaurus 16%, MeSH 19%, SNOMED CT 30%, and the UMLS 54% of the concepts in the HPO (Winnenburg and Bodenreider 2014).

The HPO is developed in the context of the Monarch Initiative (monarchinitiative.org/), whereby HPO classes are logically related to terms from other ontologies for anatomy, cell types, function (Gene Ontology), embryology, pathology, and other domains. The links provide computational definitions for HPO terms that can be used both for quality control as well as sophisticated computational phenotypic comparison. The logical links enable interoperability with numerous resources, including human genotype–phenotype resources such as OMIM (Amberger et al. 2015) and ClinVar (Landrum et al. 2014), but also those containing phenotype information on model organisms such as mouse and zebrafish (Gkoutos et al. 2009; Washington et al. 2009; Mungall et al. 2010; Köhler et al. 2013; Robinson and Webber 2014). Furthermore, human disease models that are annotated with HPO terms can be related to mouse and zebrafish models at databases including the Mouse Genome Database (Blake et al. 2014) and ZFIN (Howe et al. 2013). Integration of patient deep-phenotyping data with the landscape of both clinical and basic research informatics resources is key to effectively leveraging a much wider diversity of relevant data for the purposes of precision medicine.

A number of tools have been developed to help physicians and researchers annotate patients with HPO terms. For example, PhenoTips provides a secure, web-based interface that closely mirrors clinician workflows to facilitate the recording of phenotypic abnormalities for patients with genetic disorders, as well as a variety of other relevant information including family and medical history (Girdea et al. 2013). PhenoDB is another useful web-based tool initially developed for the Centers of Mendelian Genomics project for storing and analyzing phenotypic information from families or cohorts (Hamosh et al. 2013). Phenotypic features are hierarchically organized according to the major headings and subheadings of the Online Mendelian Inheritance in Man (OMIM) clinical synopses. The terms of PhenoDB have been mapped to HPO terms, enabling interoperability with other resources.

The HPO is being used by a number of groups in human genetics to annotate and analyze phenotypic features of patients against the background of knowledge about human diseases and animal models of disease in order to prioritize novel disease genes and perform genomic diagnostics (Riggs et al. 2012; Sifrim et al. 2013; Javed et al. 2014; Petrovski and Goldstein 2014; Robinson et al. 2014; Singleton et al. 2014; Soden et al. 2014; Zemojtel et al. 2014; Wright et al. 2015). Among the groups and projects using the HPO are the U.K. 100,000 Genomes Project (rare diseases), the Canadian CARE for RARE, PhenomeCentral (https://phenomecentral.org/), the case matching system GeneYenta (Gottlieb et al. 2015), the U.S. National Institutes of Health Undiagnosed Diseases Program and Network, and the Sanger Institute's Deciphering Developmental Disorders (DDD) (Wright et al. 2015) and DECIPHER (Firth et al. 2009) databases. Therefore, annotations of articles in *Cold Spring Harbor Molecular Case Studies* with HPO terms will open up the possibility of interlinking data with an ever-richer ecosystem of phenotypic data and sophisticated computational algorithms.

*Cold Spring Harbor Molecular Case Studies* requires authors to select HPO terms during submission that will be displayed alongside the manuscript to improve visibility. Authors should only annotate abnormal phenotypes for the case(s) described in their articles and use a precise and comprehensive set of HPO terms to maximize the ability of search engines to find their article. As the number of articles increases, researchers and physicians would be able to search for articles that describe patients with a set of phenotypic abnormalities and hopefully take advantage of semantic comparison algorithms such as the Phenomizer (Köhler et al. 2009) and not simply rely upon single-phrase matching.

Articles in *Cold Spring Harbor Molecular Case Studies* will present clinical and molecular data obtained by -omics and related approaches with the goal of elucidating disease pathogenesis and gaining insights into therapeutic strategies. Encoding the salient aspects of the clinical presentation using Human Phenotype Ontology terms will enable the articles to be searched according to phenotypic presentations, something that is currently difficult to do with standard search engines. Ontology-based indexing of articles thus represents an important step toward improving the ability of researchers and clinicians to get biomedical information that is critical for clinical care or translational research—and to realize the goal of precision medicine.

## ADDITIONAL INFORMATION

### Acknowledgments

This work was supported by grants from the Bundesministerium für Bildung und Forschung (Förderkennziffer FKZ 1315848A), the European Commission's Seventh Framework Program (SYBIL, 602300), and the National Institutes of Health (grant 5R24OD011883).

### Competing Interest Statement

The authors have declared no competing interest.
